# Repurposing the Anticancer Drug KP46 to Beat the CRAB out of Resistance: Towards an Orally Active Ga-Based Antiplantonic and Antibiofilm Agent

**DOI:** 10.3390/antibiotics14121175

**Published:** 2025-11-21

**Authors:** Guanyu Chen, LeDarius Whitley, Xiaogang Tong, Scott D. Bunge, Min-Ho Kim, Woo Shik Shin, Songping D. Huang

**Affiliations:** 1 Department of Chemistry and Biochemistry, Kent State University, Kent, OH 44240, USA; gchen10@kent.edu (G.C.); lwhitle1@kent.edu (L.W.); xtong3@kent.edu (X.T.); sbunge@kent.edu (S.D.B.); 2 Department of Biological Sciences, Kent State University, Kent, OH 44240, USA; mkim15@kent.edu; 3 Department of Pharmaceutical Sciences, College of Pharmacy and Health Sciences, Fairleigh Dickinson University, Florham Park, NJ 07932, USA

**Keywords:** antimicrobial drug repurposing, carbapenem-resistant *Acinetobacter baumannii*, Ga-based antimicrobials, biofilm inhibition by Ga

## Abstract

**Background:** Carbapenem-resistant *Acinetobacter baumannii* (CRAB) is a critical public health threat, particularly in hospital environments where treatment options are limited. Drug repurposing offers a rapid and cost-effective strategy to address antimicrobial resistance. This study evaluates KP46 (tris(8-quinolinolato)gallium(III)), an orally bioavailable gallium-based anticancer agent, for its antimicrobial potential against CRAB. **Methods:** KP46 was synthesized and characterized using spectroscopic and crystallographic techniques. Its antibacterial activity was assessed against planktonic and biofilm-associated CRAB strains, including multidrug-resistant clinical isolates. Mechanistic studies included intracellular reactive oxygen species (ROS) quantification, membrane integrity assays, and resistance development profiling. **Results:** KP46 exhibited potent antibacterial activity against both susceptible and carbapenem-resistant *A. baumannii* strains. It inhibited planktonic growth and disrupted early biofilm formation. KP46 induced intracellular oxidative stress, leading to membrane damage and cell death. Resistance development was significantly slower compared to meropenem, and KP46 retained efficacy against meropenem-resistant isolates. **Conclusions:** KP46 demonstrates dual-action antimicrobial activity and a low propensity for resistance development, positioning it as a promising candidate for repurposing against CRAB infections. These findings support further preclinical evaluation of KP46 as an orally active therapeutic agent targeting both planktonic and biofilm-associated bacterial populations. resistance development and retained efficacy against meropenem-resistant strains.

## 1. Introduction

*Acinetobacter baumannii* (AB) is a Gram-negative opportunistic pathogen notorious for its ability to acquire resistance to multiple antibiotics [1,2,3]. It is a leading cause of hospital-acquired infections, including pneumonia, bloodstream infections, and wound infections, particularly among critically ill patients [4,5]. The emergence of carbapenem-resistant *Acinetobacter baumannii* (CRAB) has further exacerbated this threat, prompting the World Health Organization (WHO) to place CRAB at the top of its 2024 Critical Priority list of drug-resistant bacteria [6].

A major contributor to the persistence and resistance of AB is its ability to form biofilm-structured bacterial communities encased in a protective extracellular matrix [7,8]. Biofilms enable the bacteria to adhere to medical devices and host tissues, shield themselves from antibiotics and immune responses, and persist in hospital environments [9]. This biofilm-associated lifestyle of AB significantly complicates treatment, as biofilm-embedded bacteria are far more resistant to conventional antibiotics than their planktonic counterparts [7,8,9].

The clinical arsenal against AB is alarmingly outdated. Most first-line antibiotics, including carbapenems, have been in use for decades, and prolonged exposure has inevitably led to the rise in resistant strains [10,11]. Ideally, combating antimicrobial resistance (AMR) requires the development of entirely new classes of antibiotics that target novel bacterial pathways. However, scientific, regulatory, and economic barriers have severely hindered progress in this area. The development of a new antibiotic typically demands over a decade of research and an investment exceeding $1.1 billion—an unsustainable model given the urgent need for new treatments [12,13].

As of now, the antibiotic development pipeline remains sparse, with only 43 candidates in clinical development and a mere 11 representing new classes [14]. This stagnation underscores the critical need for alternative strategies. One promising approach is drug repurposing—leveraging existing therapeutics with known safety profiles to address new clinical challenges. By identifying new uses for existing therapeutics with known safety profiles, this approach can significantly reduce development time and cost while addressing urgent clinical needs.

In this study, we investigate the repurposing of KP46, a gallium-based, orally bioavailable anticancer agent previously studied for the treatment of colon, lung, and bone cancers [15,16]. We hypothesized that KP46, due to its unique mechanism of action, could exhibit antimicrobial activity against multidrug-resistant CRAB. Specifically, the gallium ion (Ga^3+^), a redox-inactive ion that closely resembles ferric ion (Fe^3+^), has emerged as a promising antimicrobial agent [17,18,19,20,21,22,23,24,25,26,27,28]. By exploiting bacterial iron acquisition systems through a “Trojan horse” strategy, Ga^3+^ interferes with iron metabolism, disrupting vital cellular processes [29]. This interference inhibits both planktonic bacterial growth and biofilm formation. Unlike conventional antibiotics, the antimicrobial mode of action (MOA) offers a novel and unique approach to combating AMR. Given the urgent medical need for novel antimicrobial agents capable of targeting both planktonic and biofilm-associated bacteria, gallium-based compounds have emerged as particularly promising candidates. In this context, KP46—a tris(8-quinolinolato)gallium(III) complex with established oral bioavailability—demonstrated dual antimicrobial activity by suppressing planktonic proliferation and disrupting early biofilm development in *Acinetobacter baumannii*. These properties position KP46 as a compelling candidate for repurposing against CRAB infections, addressing a critical therapeutic gap in the management of biofilm-associated multidrug-resistant pathogens. These results reported in this article highlight the potential of drug repurposing to “beat the CRAB out of resistance” and reinvigorate the antimicrobial pipeline.

## 2. Results and Discussion

### 2.1. Synthesis and Characterization of KP46

**Figure 1:**
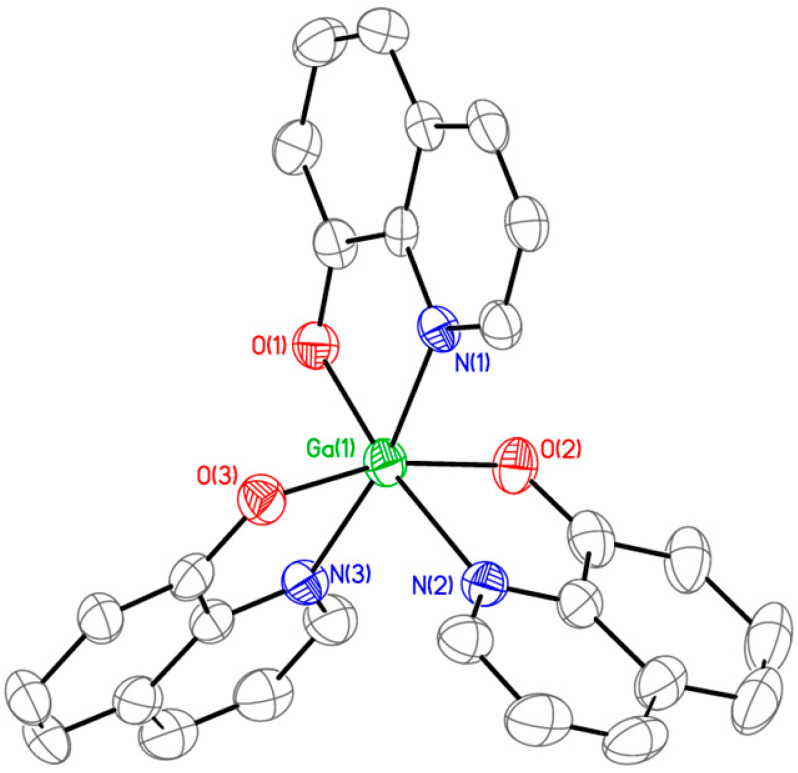
ORTEP representation of the single-crystal X-ray structure of KP46, shown with selected atom labeling. Hydrogen atoms are omitted for clarity.

An off-white precipitate was obtained by combining an aqueous solution of gallium nitrate, Ga(NO_3_)_3_, with three molar equivalents of 8-hydroxyquinoline (8HQ), which had been pre-dissolved in an ethanolic (50%) solution containing sodium carbonate (Na_2_CO_3_). The reaction mixture was stirred vigorously and refluxed at 80 °C for 3 h to promote precipitation (Scheme S1). The resulting solid was collected by filtration and washed three times with a water–ethanol mixture (50%). Comprehensive characterization by UV-Vis spectroscopy (Figure S1), FT-IR spectroscopy (Figure S2), ^1^H NMR spectroscopy (Figure S3), HPLC (Figure S4), and elemental analysis (Table S1) confirmed the identity of the product as tris(8-quinolinolato)gallium(III) with the molecular formula Ga(8HQ)_3_ and a purity exceeding 97%. To unequivocally confirm the identity of the complex, we performed single-crystal X-ray diffraction analysis on the product. Pale-yellow crystals of KP46 suitable for single-crystal X-ray diffraction analysis were obtained via vapor diffusion of hexanes into a concentrated chloroform solution. KP46 was found to crystallize in the monoclinic space group *C2/c*, with eight Ga(III) complexes per unit cell. Since the 8-hydroxyquinoline ligand is asymmetric with respect to its donor atoms, the complex can potentially exist as two stereoisomers: meridional (*mer-*) and facial (*fac-*). As shown in Figure 1, the *mer-* arrangement places two oxygen atoms and/or two nitrogen atoms opposite each other, spanning the meridian of the octahedron. Overall, KP46 exhibits metrical parameters similar to those reported in previous structural studies of the complex [30,31,32,33,34]. Notably, the Ga(III) center is fully encapsulated within the octahedral cavity formed by the lipophilic ligands, effectively shielding its ionic character. This structural feature contributes to the overall neutrality of the complex and enhances its membrane permeability.

### 2.2. Antibacterial Activity of KP46 Against Different Strains of AB Bacteria

To examine the antibacterial potential of KP46, we tested its activity against a panel of various strains of AB, including a standard laboratory strain (ATCC 19606), a meropenem-resistant *A. baumannii* strain generated from ATCC 19606 during the drug-resistance study, and seven clinical isolates obtained from the Cleveland Clinic (Table S2). These clinical isolates were previously characterized for their β-lactamase profiles and the well-known carbapenemase genes such as OXA-23, OXA-72, and OXA-82, alongside cephalosporinases (ADC variants) and, in some strains, the class A enzyme TEM-1. The combination of these resistance determinants reflects a real-world resistance scenario frequently encountered in hospital settings, where treatment options are becoming increasingly limited.

Among current treatment options, meropenem remains one of the last-resort antibiotics for multidrug-resistant Gram-negative pathogens. The broad-spectrum efficacy and favorable pharmacokinetics of meropenem have made it a cornerstone in managing severe AB infections. However, resistance to meropenem, particularly through the expression of OXA-type carbapenemases, has significantly undermined its clinical utility. According to CLSI 2024 standards [35], AB strains with meropenem MICs ≥ 8 µg/mL are considered resistant. In our strain panel, the seven Cleveland Clinic isolates surpassed this threshold, confirming their classification as carbapenem-resistant AB (CRAB).

Despite this high-level resistance, KP46 exhibited potent activity across the strain set. As summarized in Table 1, its MIC values ranged from 5 to 10 µg/mL for most clinical isolates, including those highly resistant to meropenem (MIC ≥ 32 µg/mL). Notably, isolates PR328 and PR352, both carrying OXA-23 family enzyme, were inhibited at 5 µg/mL of KP46, a concentration four to six times lower than their meropenem resistance threshold. These findings suggest that KP46 may evade the classical resistance mechanisms that limit β-lactam efficacy, likely due to its distinct Ga-based structure and non-β-lactam mode of action.

**Figure 2:**
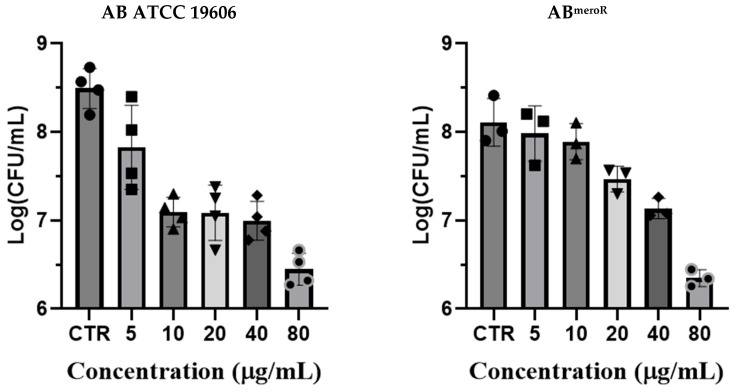
Growth inhibitory effect of KP46 on *Acinetobacter baumannii* strains ((**Left**): ATCC 19606; (**Right**): AB^meroR^). Data represent mean ± SD from three independent experiments (n = 3). CFU counts are plotted on a logarithmic scale to illustrate dose-dependent reductions in bacterial viability.

To corroborate these MIC findings, we performed colony-forming unit (CFU) reduction assays on both the ATCC 19606 reference strain and AB^meroR^. As shown in Figure 2, KP46 exhibited a dose-dependent reduction in bacterial viability, with >2-log CFU/mL reductions observed at 40 and 80 µg/mL. This bactericidal activity was once again observed in both susceptible and carbapenem-resistant backgrounds, suggesting that KP46 may bypass common resistance mechanisms associated with β-lactamase expression.

These results position KP46 as a promising repurposed candidate for the treatment of CRAB infections. Unlike β-lactam antibiotics, whose efficacy is eroded by enzyme-mediated hydrolysis, KP46 likely exerts its antimicrobial effect through a distinct, non-β-lactam mechanism that possibly involves iron mimicry and redox imbalance. The broad activity spectrum of KP46 against diverse clinical CRAB strains highlights its translational potential as an orally available agent to fill the critical therapeutic gap left by carbapenem resistance.

Earlier preclinical studies in Swiss mice reported oral LD_50_ values of 2870 mg/kg for males and 2370 mg/kg for females, indicating moderate acute toxicity. Subacute toxicity evaluations revealed that daily doses of 62.5 mg/kg were well tolerated, while higher doses (≥125 mg/kg/day) led to reduced survival and leukopenia, suggesting dose-dependent hematological toxicity [36].

Recent investigations have expanded on these findings, confirming KP46’s selective biodistribution and safety profile. A 2025 study reported that KP46 continues to demonstrate low systemic toxicity at therapeutic doses, with minimal off-target accumulation in critical organs such as the brain, lungs, and reproductive tissues. Importantly, gallium levels remained highest in bone and liver, supporting its potential for skeletal tumor targeting [37].

Overall, KP46 exhibits a favorable safety profile at clinically relevant doses and shows selective gallium accumulation in bone tissue. These properties make it a promising candidate for bone-targeted cancer therapy, although its limited absorption and tumor delivery remain challenges for further development.

### 2.3. Measurements of Intracellular ROS Generation

Previous studies on Ga-based compounds have highlighted their capability to induce intracellular reactive oxygen species (ROS) generation as part of their antimicrobial mechanism [28]. ROS production can lead to oxidative stress within bacterial cells, subsequently damaging critical cellular structures, including lipids, proteins, and nucleic acids. Based on this known property of Ga complexes, we explored whether KP46 similarly induces ROS accumulation in AB cells, contributing to its antibacterial activity.

To investigate intracellular ROS generation triggered by KP46, we treated AB bacteria with increasing concentrations of the compound (5, 10, 20, and 40 µg/mL) for 2 h. ROS levels were quantitatively assessed using the fluorescent probe 2′,7′-dichlorofluorescein diacetate (DCFH-DA), a widely used indicator of oxidative stress. Fluorescence intensity was measured and normalized against untreated control bacteria to account for variations in cell numbers and viability.

**Figure 3:**
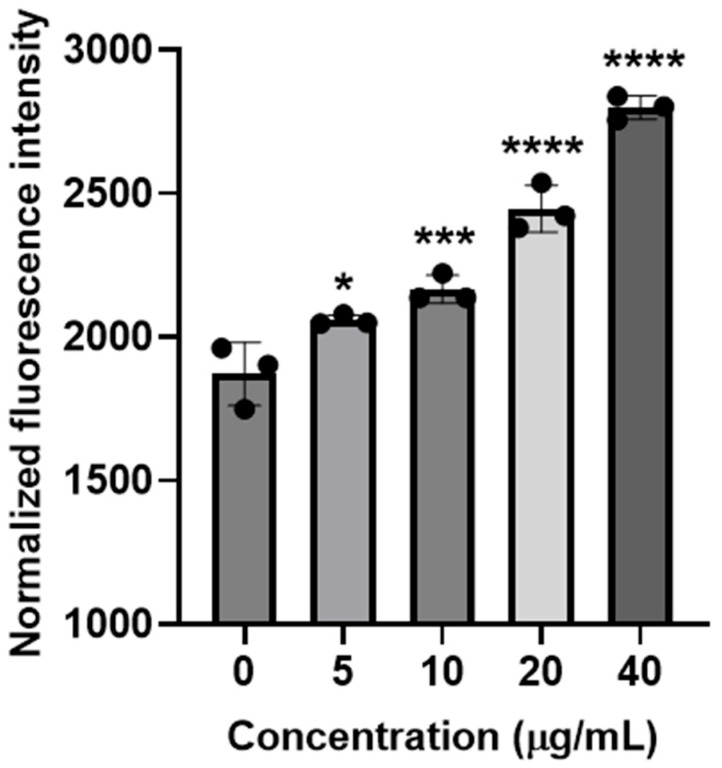
Concentration-dependent intracellular ROS generation in AB (ATCC 19606) bacteria treated with KP46. * *p* < 0.05, *** *p* < 0.001, and **** *p* < 0.0001.

As shown in Figure 3, KP46 treatment clearly resulted in a dose-dependent elevation in intracellular ROS levels within AB bacterial cells. ROS production at 40 µg/mL KP46 was significantly higher compared to lower doses (5 and 10 µg/mL). Importantly, this increase in intracellular ROS correlated with previously observed antibacterial efficacy, further strengthening the argument that oxidative damage is a primary antibacterial mode of action for KP46.

These findings collectively suggest that KP46 exerts its antimicrobial effects against CRAB primarily through the induction of intracellular oxidative stress, paralleling mechanisms observed with other Ga-based compounds. This oxidative damage pathway provides additional mechanistic insights into KP46′s potent activity, highlighting its potential as a therapeutic agent to address antibiotic-resistant bacterial infections.

### 2.4. Investigation of Anti-Biofilm Activity of KP46

To evaluate the anti-biofilm efficacy of KP46 against AB, we utilized both quantitative CFU enumeration and fluorescence-based viability imaging to assess its impact on early biofilm development. All assays were conducted under static growth conditions using both the reference strain ATCC 19606 and a meropenem-resistant *A. baumannii* strain generated from ATCC 19606 during the drug-resistance study (AB^meroR^).

**Figure 4:**
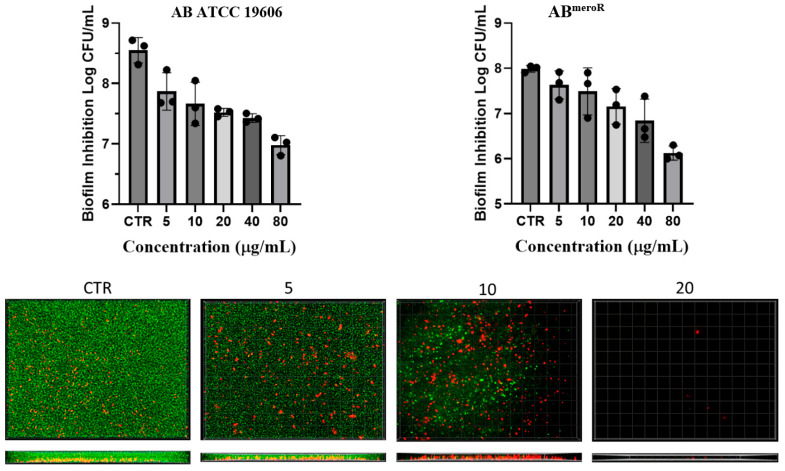
Results of quantification of biofilm inhibition activity of KP46 in AB (ATCC 19606) (**top left**) and AB^meroR^ (**top right**). Data represent mean ± SD from three independent experiments (n = 3). CFU reductions are shown on a log scale to highlight concentration-dependent inhibition. Representative fluorescence images (**bottom panels**) show Live/Dead staining (SYTO 9/PI) of biofilm-associated cells.

For the CFU-based assay, overnight bacterial cultures were diluted to 5 × 10^5^ CFU/mL in nutrient broth and incubated with increasing concentrations of KP46 in 24-well plates for 24 h, corresponding to the early phase of biofilm development. Following incubation, loosely attached planktonic cells were removed, and nascent biofilms were dislodged by vigorous pipetting in phosphate-buffered saline (PBS). Serial dilutions of the recovered cells were plated on nutrient agar, and colony-forming units (CFUs) were enumerated after 24 h. As shown in the top panels of Figure 4, KP46 markedly reduced the number of biofilm-associated viable cells in a concentration-dependent manner. At 40 µg/mL, both strains exhibited a >3-log reduction in CFU, indicating substantial inhibition of biofilm initiation and bacterial adherence.

To further assess the impact of KP46 on cell viability during early biofilm development, we conducted a live/dead bacterial staining assay using the SYTO 9/propidium iodide (PI) system. Bacterial cultures were incubated with varying concentrations of KP46 directly in chamber slides under the same conditions described above. After 24 h of static growth, the cultures were stained and visualized via fluorescence microscopy. SYTO 9 labels all bacteria with intact membranes, emitting green fluorescence, while PI selectively penetrates cells with compromised membranes, producing red fluorescence. As shown in the bottom panels of Figure 4, untreated control wells exhibited dense populations of green-fluorescent cells, indicative of vigorous bacterial growth and early biofilm formation dominated by viable cells. In contrast, KP46 treatment led to a concentration-dependent reduction in total biomass and a marked shift in fluorescence from green to red. At 20 µg/mL, only a few adherent bacteria remained, most of which were PI-positive, indicating extensive membrane damage. These findings suggest that KP46 not only inhibits bacterial proliferation and surface attachment but also significantly compromises the viability of cells initiating biofilm formation.

Together, these findings indicate that KP46 acts at the earliest stages of biofilm development to inhibit both cell attachment and survival. Its dual action of suppressing cell viability and preventing matrix establishment underscores its therapeutic value in preventing biofilm-associated AB infections, particularly in high-risk clinical environments such as those involving catheters, ventilators, or surgical implants.

### 2.5. Evaluation of Ga Resistance Development

To evaluate the risk of Ga resistance development under prolonged exposure, AB (ATCC 19606) bacteria were serially passaged for 31 consecutive days in the presence of subinhibitory concentrations (½× MIC) of either meropenem or KP46. At each 24 h interval, the minimum inhibitory concentration (MIC) was re-evaluated and expressed as fold changes relative to the initial MIC on Day 1.

**Figure 5:**
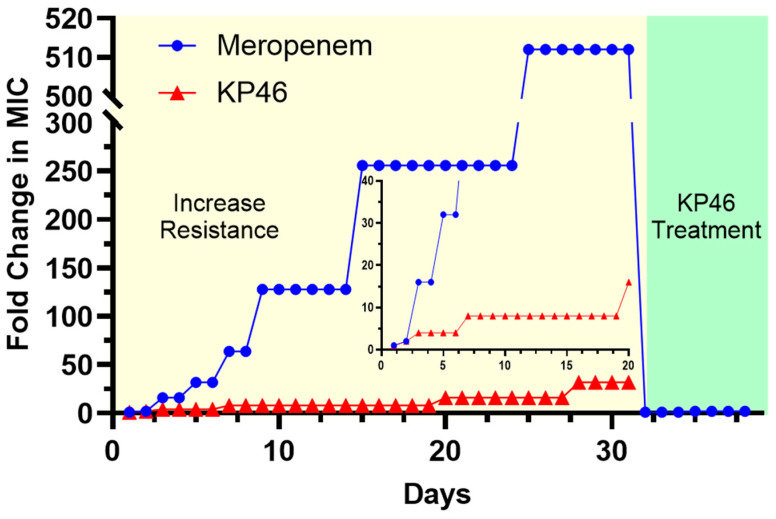
Development of resistance in *A. baumannii* ATCC 19606 during 31-day serial passaging under sub-inhibitory concentrations of KP46 or meropenem. MIC fold-change is plotted on a logarithmic scale.

As shown in Figure 5, meropenem rapidly induced high-level resistance. A 16-fold increase in MIC was observed by Day 3, followed by a sharp escalation that reached over 500-fold by Day 25. The MIC fold change remained at this elevated level through Day 31. In contrast, KP46 exhibited a much more gradual and limited increase in MIC fold change. Over the 31-day period, the MIC changed from 1-fold to a maximum of only 16-fold, with most of the increase plateauing between Day 8 and Day 20. Notably, the development of resistance to KP46 proceeded approximately 30 times more slowly than that to meropenem.

To further explore the risk of cross-resistance, we exposed the meropenem-resistant strain (Day 31) to KP46. As indicated in the green-shaded region of Figure 5, this strain remained fully susceptible to KP46, with no observable increase in KP46 MIC fold change. This result strongly suggests that KP46 retains activity even against strains with established resistance to conventional β-lactam antibiotics.

Taken together, these results underscore the low resistance potential of KP46 and its ability to maintain efficacy where standard-of-care antibiotics like meropenem fail. The minimal fold increase over time supports the hypothesis that KP46 acts through mechanisms that are not easily circumvented by traditional resistance pathways, highlighting its value as a promising therapeutic agent for combating persistent and drug-resistant AB infections.

## 3. Materials and Methods

Chemicals and reagents were obtained from commercial suppliers and used without further purification. Meropenem (≥98%), Ga(NO_3_)_3_·xH_2_O, 8-hydroxyquinoline, Na_2_CO_3_, ethanol, and DMSO were purchased from MilliporeSigma (Burlington, MA, USA). All cell-culture media and associated supplies were obtained from Thermo Fisher Scientific (Waltham, MA, USA). The laboratory strain *Acinetobacter baumannii* (ATCC 19606) was purchased from ATCC (Manassas, VA, USA). A meropenem-resistant *A. baumannii* strain was generated from ATCC 19606 during the drug-resistance study. The other seven *A. baumannii* clinical isolates were carbapenem-resistant strains obtained from the Cleveland Clinic and tested at Northeast Ohio Medical University. Details of the clinical isolates, including their resistance phenotypes, are provided in Table S2.

### 3.1. Synthesis of KP46 (i.e., Tris(8-Quinolinolato)Gallium(III))

Ga(NO_3_)_3_·xH_2_O (1.0 g) was dissolved in deionized water (25 mL). In a separate flask, 8-hydroxyquinoline (8HQ, 1.7 g) was dissolved in ethanol (50 mL) to afford a clear yellow solution. Under vigorous stirring, the aqueous gallium nitrate solution was added dropwise to the ethanolic 8HQ solution. After 10 min, 25 mL of deionized water containing Na_2_CO_3_ (0.625 g) was added dropwise to the reaction mixture under vigorous stirring. The mixture was heated to 80 °C (reflux) and maintained for 2 h. Fine yellow crystals formed during heating. The suspension was cooled to room temperature, and the solid was collected by vacuum filtration, washed with hot 50% (*v*/*v*) ethanol, and dried under vacuum at 50 °C for 48 h to give KP46 as a yellow powder (yield: 87%). The crude KP46 powder was recrystallized from a mixed solvent of chloroform and methanol. Rhombohedral, columnar orange crystals were obtained within one week by slow solvent evaporation at ambient temperature. Characterization works with the KP46 crystal. ^1^H NMR (Chloroform-d) δ 8.89 (dd, *J* = 15.1, 4.7 Hz, 3H), 8.29 (dd, *J* = 32.8, 7.9 Hz, 3H), 7.53 (t, *J* = 8.0 Hz, 3H), 7.50–7.35 (m, 3H), 7.27–7.04 (m, 6H). Elemental analysis, calculated C% 64.58, H% 3.61, N% 8.37; Found C% 63.77, H% 3.82, N% 8.12. The HPLC trace of KP46 shows that the product is 99.0% pure.

### 3.2. Single-Crystal X-Ray Diffraction Analysis

A suitable single crystal for X-ray diffraction studies was obtained for compound KP46. Crystal data for the compound was obtained by mounting a pale-yellow crystal onto a thin glass fiber from a pool of Fluorolube^TM^ and immediately placing it under a liquid N_2_-cooled stream on a Bruker AXS diffractometer (Billerica, MA, USA) upgraded with an APEX II CCD detector. The radiation used is graphite monochromatized Mo Kα radiation (λ = 0.7107 Å). The lattice parameters are optimized from a least-squares calculation on carefully centered reflections. Lattice determination, data collection, structure refinement, scaling, and data reduction were carried out using APEX6 Version 2024.9-0 software package [38,39]. The data were corrected for absorption using the SCALE program within the APEX6 software package [40,41]. The structure was solved using SHELXT-2019. This procedure yielded the Ga and a number of the C, N, and O atoms. Subsequent Fourier synthesis yielded the remaining atom positions. The hydrogen atoms are fixed in positions of ideal geometry (riding model) and refined within OLEX2-1.5. The final refinement of each compound, which included anisotropic thermal parameters on all non-hydrogen atoms, was performed using OLEX2-1.5. The crystal data for compound KP46 are given in Table S3.

### 3.3. Evaluation of Minimum Inhibitory Concentrations (MICs)

Minimum inhibitory concentrations (MICs) of KP46 against nine *Acinetobacter baumannii* strains were determined by the standard broth microdilution procedure (CLSI 2024 standards) as described previously [28,35]. Briefly, bacterial suspensions were adjusted to 1 × 10^6^ CFU mL^−1^ in Nutrient Broth (NB) and exposed to a concentration series of KP46 crystal DMSO solution (1.25~160 μg/mL, DMSO 1% *v*/*v*). Aliquots (200 μL) were dispensed into sterile 96-well microplates and incubated at 37 °C for 18 h under static conditions. The MIC was defined as the lowest KP46 concentration yielding no visible growth in the well.

### 3.4. Colony-Forming Unit (CFU) Reduction Assays

A single colony was inoculated into 5 mL NB and cultured at 37 °C, 180 rpm for 18 h to obtain an overnight culture (≈1 × 10^9^ CFU mL^−1^). For treatment, 1 μL of the overnight culture was mixed with 990 μL fresh NB and 10 μL of KP46 stock solution from 0~16 mg/mL in DMSO (final DMSO 1% *v*/*v*) to achieve the indicated KP46 concentrations in a total volume of 1.0 mL. Suspensions were incubated in an incushaker at 37 °C, 180 rpm for 18 h. Samples were then serially diluted and plated on nutrient agar; colonies were enumerated after 24 h at 37 °C to calculate CFU mL^−1^. All measurements were performed in triplicate.

### 3.5. Determination of Intracellular ROS Generation

Intracellular reactive oxygen species (ROS) were quantified following our previously reported protocol with minor adaptations [28,42]. Briefly, overnight cultures of *A. baumannii* were harvested by centrifugation (3750 rpm, 7 min), resuspended in fresh NB (400 μL), and distributed as 100 μL aliquots into tubes containing 890 μL NB and 10 μL KP46 stock (final DMSO 1% *v*/*v*). After incubation at 37 °C for 2 h with gentle agitation, cells were collected, washed once with HBSS (1×), and incubated with 20 μM DCFH-DA at 37 °C for 30 min in the dark. The fluorescence of the resulting suspensions was recorded on a SpectraMax M4 microplate reader (Molecular Devices, Sunnyvale, CA, USA) at Ex/Em = 497/529 nm.

### 3.6. In Vitro Evaluations of Resistance Development

Resistance trajectories were assessed by daily serial passaging under sub-inhibitory drug pressure, adapted from published experimental-evolution workflows [28,43]. *A. baumannii* cultures were exposed to KP46 or meropenem for 30 consecutive days, and MIC changes were monitored over time. Each day, bacteria were inoculated into fresh NB containing ½× MIC (day-specific) of the respective drug and incubated at 37 °C for 18 h. From the growth condition with the highest drug level that still supported proliferation, cells were collected, and the MIC for that passage was determined. Results are presented as fold-change in MIC relative to the initial MIC for each drug across passages.

### 3.7. Investigation of Antibiofilm Activity of KP46

Overnight cultures were diluted in NB to 1 × 10^6^ CFU mL^−1^. Aliquots of 500 μL bacterial suspension containing KP46 at the indicated concentrations were distributed into sterile 24-well plates and incubated statically at 37 °C for 24 h to allow biofilm formation. Spent medium was aspirated, and biofilms were dislodged by vigorous pipetting in 500 μL PBS, followed by serial dilution. Fifty microliters of each dilution were spread on nutrient agar. Colony counts obtained after 24 h at 37 °C were used to calculate log_10_ reductions in viable biofilm-associated cells relative to untreated controls. All experiments were conducted in triplicate.

### 3.8. SYTO 9/Propidium Iodide Live–Dead Staining

For fluorescence imaging, an overnight culture was adjusted to 1 × 10^6^ CFU mL^−1^ and dispensed at 200 μL per well into sterile 8-well chambered coverslips containing KP46 at the indicated concentrations. After static incubation at 37 °C for 24 h, SYTO 9/propidium iodide (LIVE/DEAD™ BacLight™ Bacterial Viability Kit, Thermo Fisher Scientific) was added (20 μL per well) and the plate was kept at room temperature in the dark for 1 h. Images were acquired on an Olympus IX81 inverted fluorescence microscope (Shinjuku-ku, Japan) using FITC/TRITC filter sets with identical exposure settings across conditions.

### 3.9. Statistical Analysis

All in vitro experiments were performed in triplicate (n = 3) unless otherwise specified. Statistical analyses were conducted in GraphPad Prism (version 8.0). Data are reported as mean ± standard deviation (SD). For two-group comparisons, a two-tailed unpaired Student’s *t*-test was used. For comparisons among multiple groups, one-way analysis of variance (ANOVA) followed by Holm–Šídák’s multiple-comparisons test was applied. Statistical significance was set at *p* < 0.05, with significance levels denoted as * *p* < 0.05, ** *p* < 0.01, *** *p* < 0.001, and **** *p* < 0.0001 considered statistically significant.

## 4. Conclusions

In conclusion, our study demonstrates that KP46, a Ga-based anticancer drug with established oral bioavailability and safety, exhibits potent antimicrobial activity against *Acinetobacter baumannii*, including carbapenem-resistant clinical isolates. KP46 effectively inhibits both planktonic growth and early biofilm formation, disrupts bacterial viability through oxidative stress, and shows minimal risk of resistance development. These findings underscore the therapeutic potential of KP46 as a repurposed antimicrobial agent and exemplify the broader promise of drug repurposing to address the critical challenge of multidrug-resistant bacterial infections. This work lays the foundation for further preclinical and clinical evaluation of KP46 as a novel treatment option for CRAB and other biofilm-associated pathogens.

## Figures and Tables

**Table 1:** MIC values of KP46 against lab and clinic isolate *Acinetobacter baumannii* strains in comparison with meropenem.

Bacterial Strains		KP46		Meropenem	
		µM	µg/mL	µM	µg/mL
Lab Strains	ATCC 19606	20	10	2.5	1
	AB^meroR^	40	20	80	32
Clinic isolate Strains	PR 328	10	5	80	128
	PR 336	20	10	80	128
	PR 347	20	10	40	128
	PR 352	10	5	>80	>128
	PR 376	10	5	>80	>128
	PR 315	20	10	10	32
	PR 380	10	5	>80	>128

## Data Availability

The original contributions presented in this study are included in the article/supplementary material. Further inquiries can be directed to the corresponding author(s). Crystallographic data for the structure has been deposited with the Cambridge Crystallographic Data Centre as supplementary publication no: CCDC 2495708. Copies of the data can be obtained, free of charge on application to CCDC, 12 Union Road, Cambridge CB2 1EZ, UK (fax, +44-(0)1223-336033; or e-mail, deposit@ccdc.cam.ac.uk).

## Supplementary Materials

The following supporting information can be downloaded at: https://www.mdpi.com/article/10.3390/antibiotics14121175/s1, Scheme S1. Synthesis of KP-46 in 50% ethanol. Figure S1. UV-vis spectra of KP-46 and 8-hydroxyquinoline (8HQ) in DMSO/ethanol mixture. Figure S2. FT-IR spectra of KP-46 and 8-hydroxyquinoline (8HQ). Figure S3. The ^1^H NMR spectra of 8-hydroxyquinoline. Figure S4. The HPLC trace of KP-46 shows that the product is 99.0% pure. Table S1. Elemental analysis results of C_27_H_18_N_3_O_3_Ga(CH_3_OH). Table S2. Cleveland Clinic isolated *Acinetobacter baumannii*’s type of bla gene and classes. Table S3. Crystal data and structure refinement for GC_KP_46_10_07_25_0m_a.
