# Supplementary material for: Repurposing the Anticancer Drug KP46 to Beat the CRAB out of Resistance: Towards an Orally Active Ga-Based Antiplantonic and Antibiofilm Agent
Source: Antibiotics (Basel). 2025 Nov 21;14(12):1175. doi: 10.3390/antibiotics14121175 (PMC12729858; doi:10.3390/antibiotics14121175)
Supplement: Supplementary file 1 [file antibiotics-14-01175-s001.zip › antibiotics-3908869-supplementary.pdf]

Repurposing the Anticancer Drug KP-46 to Beat the CRAB Out of Resistance: Towards an Orally Active Ga-based Antiplantonic and Antibiofilm Agent

Guanyu Chen[1], LeDarius Whitley[1], Xiaogang Tong[1], Scott D. Bunge[1], Min-Ho Kim[2], Woo Shik Shin[*3] and Songping D. Huang[*1]

**Corresponding author's email: shuang1@kent.edu.*

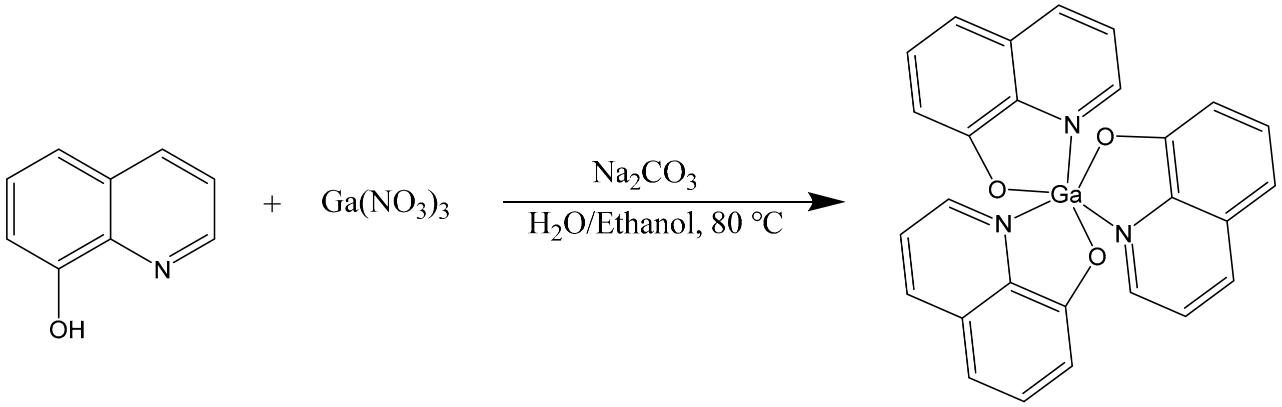

Scheme S1. Synthesis of KP-46 in 50% ethanol

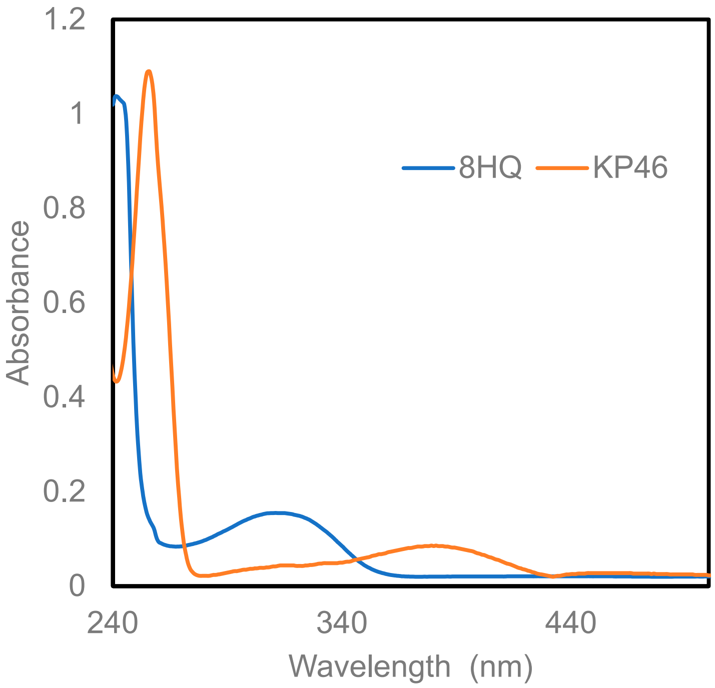

Figure S1. UV-vis spectra of KP-46 and 8-hydroxyquinoline (8HQ) in DMSO/ethanol mixture. The significant red-shift in the absorption maximum from ~245 nm in 8HQ to 257 nm in KP46, along with the shift in a broad absorption peak from ~317 nm in 8HQ to 386 nm, indicates coordination of Ga(III) to the 8HQ ligand molecules. indicates coordination of Ga(III) to the8HQ ligand molecules.

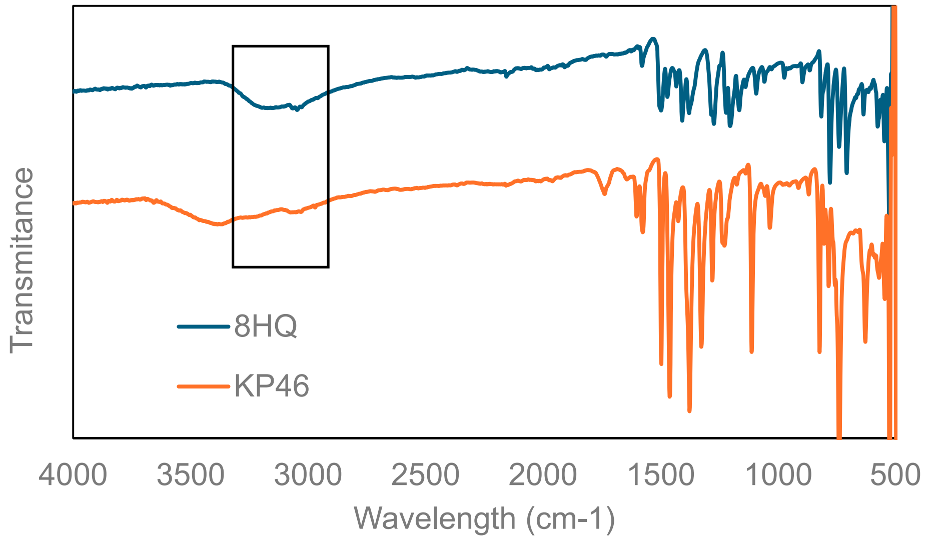

Figure S2. FT-IR spectra of KP46 (red) and 8-hydroxyquinoline (8HQ, blue). The disappearance of the characteristic O–H stretching vibration of 8HQ around 3100–3300 cm⁻¹ in the KP46 spectrum indicates deprotonation of the hydroxyl group upon coordination of Ga(III) to the 8HQ ligand.

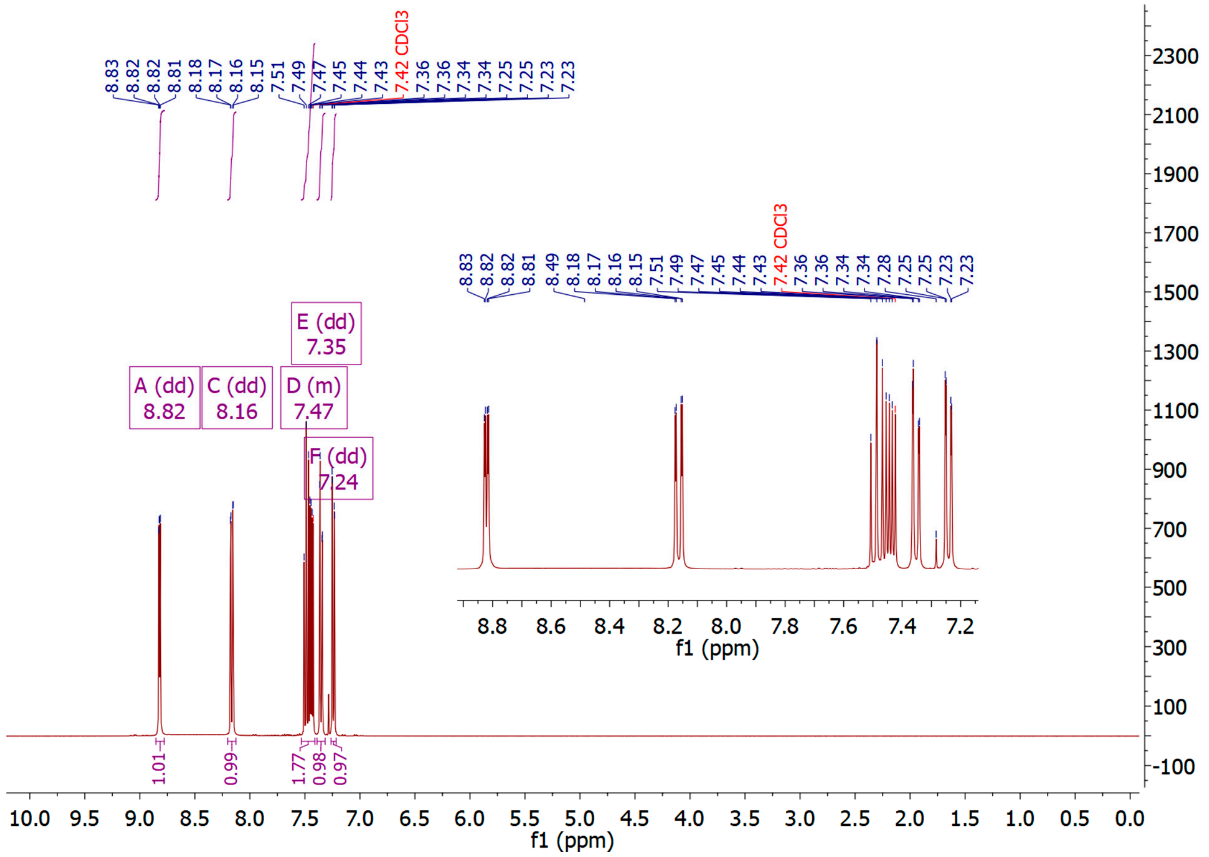

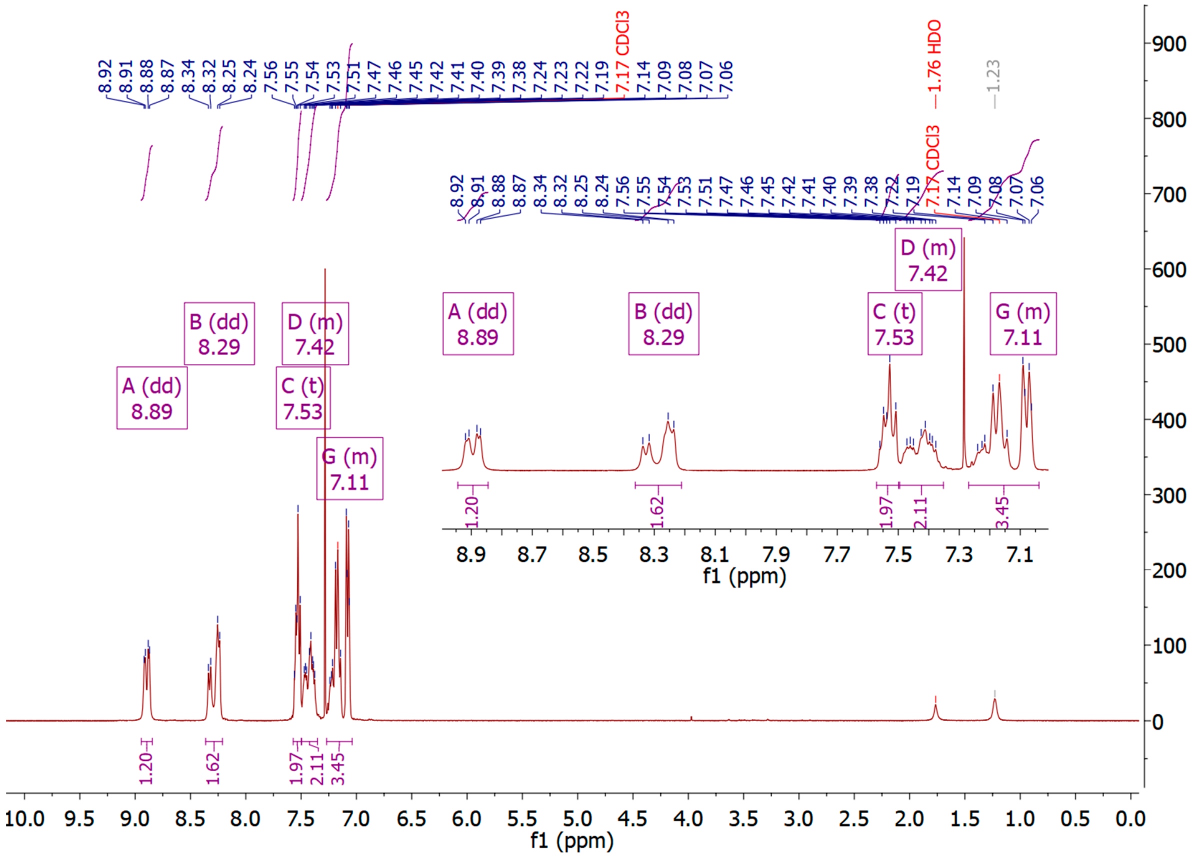

Figure S3. The ^{1}H NMR spectra of 8-hydroxyquinoline (TOP, 400 MHz, Chloroform-d) δ 8.82 (dd, J = 4.2, 1.6 Hz, 1H), 8.16 (dd, J = 8.3, 1.6 Hz, 1H), 7.53 – 7.41 (m, 1H), 7.35 (dd, J = 8.3, 1.2 Hz, 1H), 7.24 (dd, J = 7.6, 1.2 Hz, 2H) and KP-46 (Bottom, 400 MHz, Chloroform-d) δ 8.89 (dd, J = 15.1, 4.7 Hz, 3H), 8.29 (dd, J = 32.8, 7.9 Hz, 3H), 7.53 (t, J = 8.0 Hz, 3H), 7.50 – 7.35 (m, 3H), 7.27 – 7.04 (m, 6H).

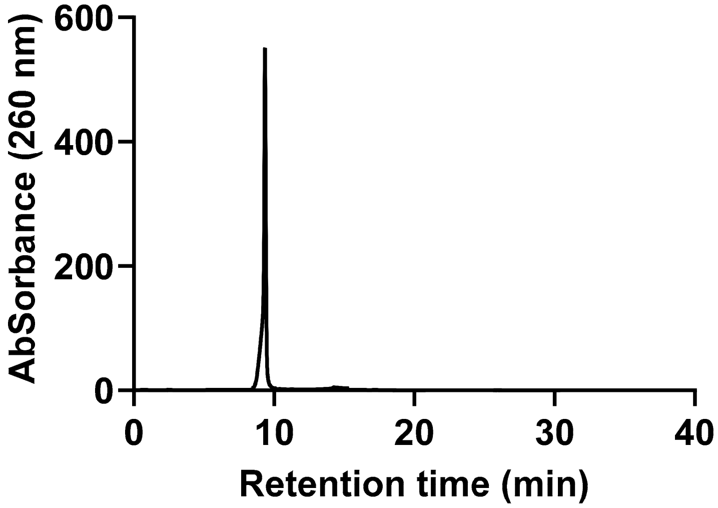

Figure S4. The HPLC trace of KP-46 shows that the product is 99.0 % pure. Gradient: 0 min 5% B, 5 min 5% B, 10 min 85% B, 15 min 90% B, 20 min 98% B, 30 min 5% B (Solvent A is 0.1% TFA aqueous solution and B is acetonitrile).

Table S1. Elemental analysis results of $C_{27}H_{18}N_3O_3Ga$

Elements	Calculated %	Experimental %
Carbon (C)	64.58	63.77
Hydrogen(H)	3.61	3.82
Nitrogen (N)	8.37	8.12

Table S2. Cleveland clinic isolated *Acinetobacter baumannii*'s type of *bla* gene and classes.

	Acinetobacter baumannii	*bla gene*	*Class*
AB1	PR309 ♠	TEM-1	A
	PR319 ♠	ADC-30, OXA-51, OXA-23, OXA-72	C,D
AB2	PR328 ♠	ADC-33, OXA-82, OXA-23	C,D
	PR336 ♠	OXA-939, ADC-159	C,D
	PR347 ♠	OXA-66, OXA-72 partial, ADC-30	C,D
AB3	PR352 ♠	OXA-23, OXA-66, ADC-30, TEM-1	A,C,D
	PR372 ♠	OXA-23, OXA-82, ADC-33	C,D
	PR376 ♠	OXA-23, OXA-66, ADC-162, TEM-1	A,C,D
	PR380 ♠	OXA-23, OXA-66, ADC-30, TEM-1	A,C,D
	PR315 ♠	OXA-121, ADC163	C,D

Table S3. Crystal data and structure refinement for GC_KP_46_10_07_25_0m_a.

Identification code	GC_KP_46_10_07_25_0m_a
Empirical formula	$C_{29}H_{28}Cl_6GaN_3O_7$
Formula weight	812.96
Temperature/K	224.99
Crystal system	monoclinic
Space group	C2/c
a/Å	27.529(9)
b/Å	15.863(5)
c/Å	16.203(5)
α/°	90
β/°	103.949(4)
γ/°	90
Volume/Å^3	6867(4)
Z	8
ρ_{calc}g/cm^3	1.573
μ/mm^{-1}	1.317
F(000)	3296.0
Crystal size/mm^3	0.55 × 0.05 × 0.05
Radiation	MoKα (λ = 0.71073)
2Θ range for data collection/°	3.704 to 52.17
Index ranges	-34 ≤ h ≤ 33, -19 ≤ k ≤ 15, -19 ≤ l ≤ 12
Reflections collected	21091
